# Lacinilene C 7-methyl ether

**DOI:** 10.1107/S1600536813021430

**Published:** 2013-08-07

**Authors:** Vyacheslav V. Uzbekov, Samat A. Talipov, Bakhtiyar T. Ibragimov, Robert D. Stipanovic, Alois A. Bell

**Affiliations:** a A.S. Sadykov Institute of Bioorganic Chemistry, Academy of Sciences of Uzbekistan, Mirzo Ulugbek str. 83, Tashkent 100125, Uzbekistan; b Southern Plains Agricultural Research Center, Agricultural Research Service, USDA, College Station, TX 77845, USA

## Abstract

The title compound, C_16_H_20_O_3_ [systematic name: 1-hy­droxy-7-meth­oxy-1,6-dimethyl-4-(propan-2-yl)naphthalen-2(1*H*)-one], is a sesquiterpene isolated from foliar tissues of the cotton plant and is of inter­est with respect to its anti­bacterial properties. Its phenyl ring is ideally planar, and the maximum of deviation in the second ring is 0.386 (3) Å. The hy­droxy group and the methyl group are oriented in an equatorial fashion and axial, respectively, to the second ring. In the crystal, inversion dimers are formed through pairs of O—H⋯O hydrogen bonds. Weak C—H⋯O hydrogen bonds link the dimers into columns along the *c* axis. These columns form a crystal structure with a crystal packing factor of 0.66.

## Related literature

For the original isolation from *Ulmus laciniata* Mayr and proposed structure, see: Suzuki *et al.* (1972 ▶). For isolation from cotton bracts (*Gossypium*), identification and structure definition, see: Stipanovic *et al.* (1975 ▶, 1981 ▶). For information on the biological activity, see: Essenberg *et al.* (1982 ▶). For biosynthetic studies, see: Stipanovic *et al.* (1981 ▶); Essenberg *et al.* (1985 ▶).
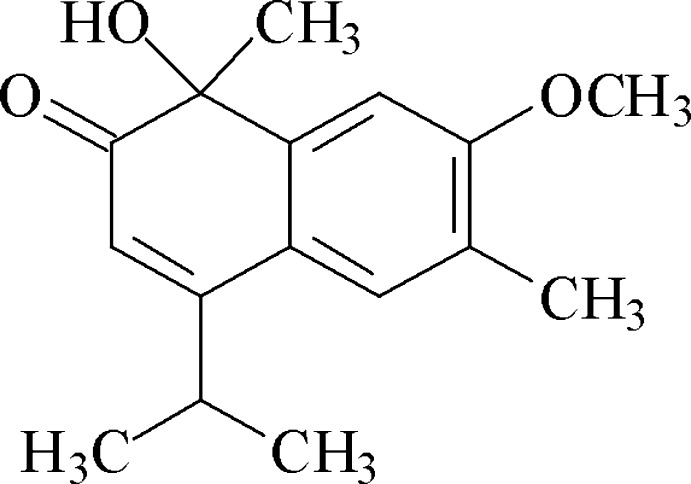


## Experimental

### 

#### Crystal data

C_16_H_20_O_3_
*M* *_r_* = 260.32
Triclinic, 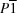

*a* = 8.285 (2) Å
*b* = 8.987 (2) Å
*c* = 10.665 (3) Å
α = 68.58 (2)°
β = 78.95 (2)°
γ = 88.87 (2)°
*V* = 724.4 (3) Å^3^
*Z* = 2
Cu *K*α radiation
μ = 0.65 mm^−1^
*T* = 295 K
0.34 × 0.27 × 0.20 mm

#### Data collection

Oxford Diffraction Xcalibur Ruby CCD diffractometer
Absorption correction: multi-scan (*CrysAlis PRO*; Oxford Diffraction, 2009 ▶) *T*_min_ = 0.809, *T*_max_ = 0.878
6210 measured reflections
2927 independent reflections
1928 reflections with *I* > 2σ(*I*)
*R*_int_ = 0.030

#### Refinement

*R*[*F* ^2^ > 2σ(*F* ^2^)] = 0.051
*wR*(*F* ^2^) = 0.171
*S* = 1.06
2927 reflections
182 parameters
H atoms treated by a mixture of independent and constrained refinement
Δρ_max_ = 0.18 e Å^−3^
Δρ_min_ = −0.18 e Å^−3^

### 

Data collection: *CrysAlis PRO* (Oxford Diffraction, 2009 ▶); cell refinement: *CrysAlis PRO*; data reduction: *CrysAlis RED* (Oxford Diffraction, 2009 ▶); program(s) used to solve structure: *SHELXS97* (Sheldrick, 2008 ▶); program(s) used to refine structure: *SHELXL97* (Sheldrick, 2008 ▶); molecular graphics: *XP* in *SHELXTL* (Sheldrick, 2008 ▶); software used to prepare material for publication: *SHELXL97*.

## Supplementary Material

Crystal structure: contains datablock(s) I, exp1290. DOI: 10.1107/S1600536813021430/rk2406sup1.cif

Structure factors: contains datablock(s) I. DOI: 10.1107/S1600536813021430/rk2406Isup2.hkl

Supplementary material file. DOI: 10.1107/S1600536813021430/rk2406Isup3.cml

Additional supplementary materials:  crystallographic information; 3D view; checkCIF report

## Figures and Tables

**Table 1:** Hydrogen-bond geometry (Å, °)

*D*—H⋯*A*	*D*—H	H⋯*A*	*D*⋯*A*	*D*—H⋯*A*
O3—H3⋯O2^i^	0.87 (3)	2.08 (3)	2.892 (2)	156.3
C13—H13*B*⋯O2^ii^	0.96	2.51	3.467 (2)	177

Symmetry codes: (i) 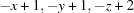; (ii) 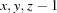.

**Fig. 1.**
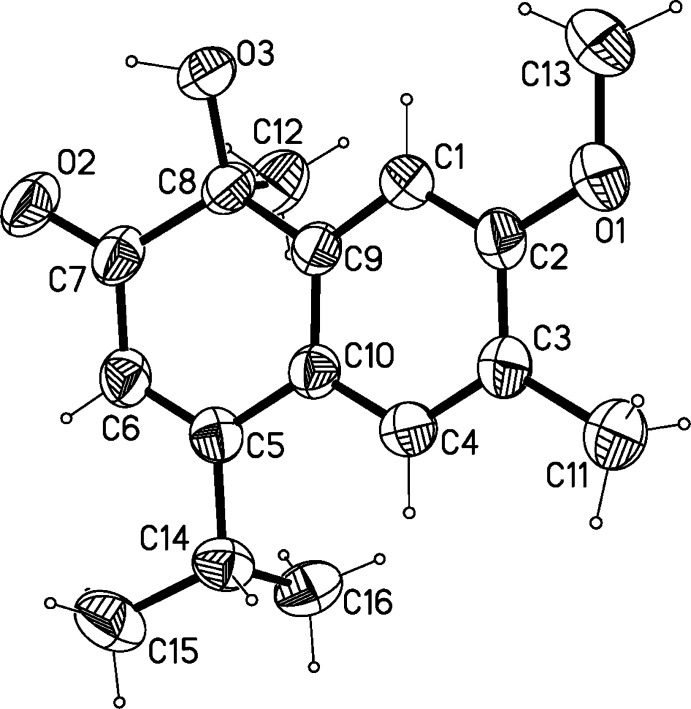
The molecular structure of title compound, with the atom–numbering scheme. Displacement ellipsoids are drawn at the 50% probability level. H atoms are presented as a small spheres of arbitrary radius.

**Fig. 2.**
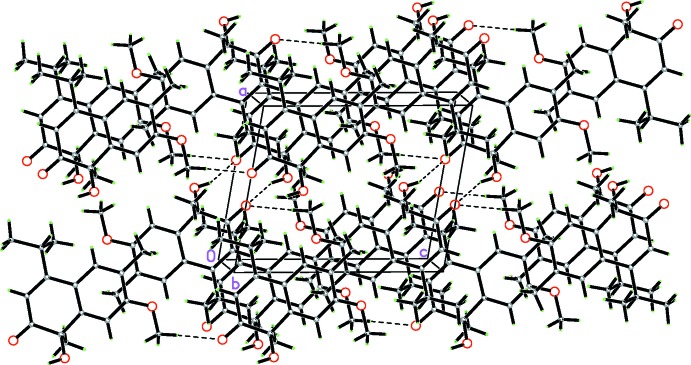
A packing diagram for title compound.

## supplementary crystallographic information

### 1. Comment

The title compound, C_16_H_20_O_3_, *LCME* is a sesquiterpene isolated from foliar tissues of the cotton plant. Its biosynthesis is induced in response to infection by the bacterial plant pathogen, *Xanthomonas campestris* pv. *malvacearum*; the latter is the causal agent of bacterial blight, and *LCME* exhibits antibacterial activity against this pathogen. *LCME* was originally isolated from *Ulmus laciniata* Mayr (Suzuki *et al.*, 1972), but the proposed structure was incorrect. Subsequently, it was isolated together with lacinilene C from frost–killed cotton bracts (*Gossypium* species) and its structure was revised (Stipanovic *et al.*, 1975). *LCME* is produced by autoxidation of 2–hydroxy–7–methoxycadalene, which also occurs in cotton plant foliage (Stipanovic *et al.*, 1981). The biosynthesis was first elucidated by Essenberg *et al.* (1985). However, lacinilene C (the un–methylated derivative of lacinilene C 7–methyl ether) isolated from cotton tissues is optically active, which indicates it is the product of enzymatic transformation of 2,7–dihydroxycadalene (Essenberg *et al.*, 1982). The conformation of the title molecule and numbering scheme of atoms is shown in Fig. 1. The atoms of the phenyl ring (C1–C4/C9/C10) are ideal planar with a r.m.s. = 0.0085 Å. In the second ring, the atoms C5–C7/C9/C10 lie in an ideal plane with a r.m.s. = 0.0313 Å, and the deviation from planarity of atom C8 is equal to 0.386 (3) Å. The dihedral angle between these planes is equal to 170.2 (1)°. The hydroxy group O3 and the methyl group C12 are oriented equatorially and axially to the second ring, respectively. In the crystal structure, the centrosymmetric dimers are formed through pairs of O3—H3···O2^i^ classical hydrogen bonds. Weak non–classical hydrogen bonds between C13—H13B···O2^ii^ of the centrosymmetric dimers associate into columns by translation along the *c* axis (Fig. 2). Symmetry codes: (i) -*x*+1,-*y*+1,-*z*+2; (ii) *x*, *y*, *z*-1. The columns form a crystal structure with a packing factor 0.66.

### 2. Experimental

The title compound was isolated from frost–killed cotton bracts (*Gossypium* species) by extraction and silica gel *LC* procedures as previously described (Stipanovic *et al.*, 1981). For achieving separation of the closely related compounds, the partially purified fraction was further chromatographed by consecutive injections on semi–preparative *RP–HPLC* column (Agilent 1100 HPLC system; Zorbax Eclipse XDB C8 column 9.4× 250 mm, 5µm; Agilent Technologies Inc, USA). The column was eluted using a linear gradient of H_2_O (*A*) /CH_3_OH (*B*) (*HPLC* grade, Sigma–Aldrich, DE) from 60 to 90% *B* for 30 minutes at a flow rate of 3 ml/min with the following segment of 100% *B* within 5 minutes and eluates were monitored at 254 nm. Crystals were obtained by slow evaporation of the *HPLC* eluent, and the most appropriate for *X*-ray diffraction were collected (m.p. 57–60°C).

### 3. Refinement

All H atoms were placed in geometrically idealized positions C—H = 0.98Å for methine H, C—H = 0.96Å for methyl H and C—H = 0.93Å for aromatic H and treated as riding on their parent atoms, with *U*_iso_(H) = 1.5*U*_eq_(C) for methyl H; *U*_iso_(H) = 1.2*U*_eq_(C) for aromatic and methine H. The H atom of hydroxy group was refined freely.

### Crystal data

**Table:**

C_16_H_20_O_3_	*Z* = 2
*M**_r_* = 260.32	*F*(000) = 280
Triclinic, *P*1	*D*_x_ = 1.194 Mg m^−^^3^
*a* = 8.285 (2) Å	Cu *K*α radiation, λ = 1.54184 Å
*b* = 8.987 (2) Å	Cell parameters from 602 reflections
*c* = 10.665 (3) Å	θ = 4.5–77.4°
α = 68.58 (2)°	µ = 0.65 mm^−^^1^
β = 78.95 (2)°	*T* = 295 K
γ = 88.87 (2)°	Block, white
*V* = 724.4 (3) Å^3^	0.34 × 0.27 × 0.20 mm

### Data collection

**Table:**

Oxford Diffraction Xcalibur Ruby CCD diffractometer	2927 independent reflections
Radiation source: fine–focus sealed tube	1928 reflections with *I* > 2σ(*I*)
Graphite monochromator	*R*_int_ = 0.030
ω scans	θ_max_ = 75.9°, θ_min_ = 4.5°
Absorption correction: multi-scan (*CrysAlis PRO*; Oxford Diffraction, 2009)	*h* = −9→10
*T*_min_ = 0.809, *T*_max_ = 0.878	*k* = −10→11
6210 measured reflections	*l* = −13→13

### Refinement

**Table:**

Refinement on *F*^2^	Secondary atom site location: difference Fourier map
Least-squares matrix: full	Hydrogen site location: inferred from neighbouring sites
*R*[*F*^2^ > 2σ(*F*^2^)] = 0.051	H atoms treated by a mixture of independent and constrained refinement
*wR*(*F*^2^) = 0.171	*w* = 1/[σ^2^(*F*_o_^2^) + (0.0933*P*)^2^ + 0.0437*P*] where *P* = (*F*_o_^2^ + 2*F*_c_^2^)/3
*S* = 1.06	(Δ/σ)_max_ < 0.001
2927 reflections	Δρ_max_ = 0.18 e Å^−^^3^
182 parameters	Δρ_min_ = −0.18 e Å^−^^3^
0 restraints	Extinction correction: *SHELXL97* (Sheldrick, 2008), Fc^*^=kFc[1+0.001xFc^2^λ^3^/sin(2θ)]^-1/4^
Primary atom site location: structure-invariant direct methods	Extinction coefficient: 0.008 (2)

### Special details

**Table:**

Geometry. All s.u.'s (except the s.u. in the dihedral angle between two l.s. planes) are estimated using the full covariance matrix. The cell s.u.'s are taken into account individually in the estimation of s.u.'s in distances, angles and torsion angles; correlations between s.u.'s in cell parameters are only used when they are defined by crystal symmetry. An approximate (isotropic) treatment of cell s.u.'s is used for estimating s.u.'s involving l.s. planes.
Refinement. Refinement of *F*^2^ against ALL reflections. The weighted *R*–factor *wR* and goodness of fit *S* are based on *F*^2^, conventional *R*–factors *R* are based on *F*, with *F* set to zero for negative *F*^2^. The threshold expression of *F*^2^ > 2σ(*F*^2^) is used only for calculating *R*–factors(gt) *etc*. and is not relevant to the choice of reflections for refinement. *R*–factors based on *F*^2^ are statistically about twice as large as those based on *F*, and *R*–factors based on ALL data will be even larger.

### Fractional atomic coordinates and isotropic or equivalent isotropic displacement parameters (Å^2^)

**Table:**

	*x*	*y*	*z*	*U*_iso_*/*U*_eq_	
O1	0.17788 (19)	0.7003 (2)	0.39017 (14)	0.0736 (5)	
O2	0.3720 (2)	0.6105 (2)	1.03293 (16)	0.0881 (6)	
O3	0.48189 (19)	0.6395 (2)	0.76954 (16)	0.0745 (5)	
C1	0.2611 (2)	0.7114 (2)	0.59257 (18)	0.0557 (5)	
H1	0.3710	0.7002	0.5588	0.067*	
C2	0.1443 (3)	0.7162 (2)	0.51528 (18)	0.0564 (5)	
C3	−0.0215 (3)	0.7362 (3)	0.56220 (19)	0.0587 (5)	
C4	−0.0648 (2)	0.7455 (2)	0.69137 (19)	0.0562 (5)	
H4	−0.1747	0.7574	0.7244	0.067*	
C5	0.0011 (2)	0.7369 (2)	0.91560 (18)	0.0507 (5)	
C6	0.1106 (3)	0.7009 (2)	0.99895 (19)	0.0587 (5)	
H6	0.0762	0.6946	1.0891	0.070*	
C7	0.2793 (3)	0.6715 (2)	0.9538 (2)	0.0600 (5)	
C8	0.3471 (2)	0.7286 (2)	0.79945 (19)	0.0553 (5)	
C9	0.2154 (2)	0.7232 (2)	0.72078 (17)	0.0491 (4)	
C10	0.0498 (2)	0.7378 (2)	0.77398 (17)	0.0488 (4)	
C11	−0.1470 (3)	0.7475 (4)	0.4749 (2)	0.0851 (8)	
H11B	−0.1295	0.8479	0.3985	0.128*	
H11A	−0.2555	0.7405	0.5288	0.128*	
H11C	−0.1361	0.6613	0.4415	0.128*	
C12	0.4083 (3)	0.9043 (3)	0.7538 (3)	0.0790 (7)	
H12A	0.4528	0.9453	0.6569	0.119*	
H12B	0.4922	0.9110	0.8026	0.119*	
H12C	0.3180	0.9662	0.7735	0.119*	
C13	0.3426 (3)	0.6738 (3)	0.3381 (2)	0.0823 (7)	
H13B	0.3478	0.6595	0.2526	0.123*	
H13C	0.3780	0.5796	0.4030	0.123*	
H13A	0.4132	0.7644	0.3237	0.123*	
C14	−0.1737 (2)	0.7743 (2)	0.9643 (2)	0.0574 (5)	
H14	−0.2483	0.7155	0.9353	0.069*	
C15	−0.2245 (3)	0.7260 (3)	1.1202 (2)	0.0831 (7)	
H15A	−0.2059	0.6148	1.1644	0.125*	
H15C	−0.3391	0.7434	1.1438	0.125*	
H15B	−0.1602	0.7895	1.1501	0.125*	
C16	−0.1950 (3)	0.9525 (3)	0.8936 (3)	0.0731 (6)	
H16C	−0.1205	1.0129	0.9180	0.110*	
H16A	−0.3062	0.9764	0.9224	0.110*	
H16B	−0.1715	0.9804	0.7958	0.110*	
H3	0.506 (4)	0.574 (4)	0.846 (3)	0.116 (11)*	

### Atomic displacement parameters (Å^2^)

**Table:**

	*U*^11^	*U*^22^	*U*^33^	*U*^12^	*U*^13^	*U*^23^
O1	0.0766 (10)	0.1039 (12)	0.0479 (7)	0.0129 (9)	−0.0140 (7)	−0.0365 (8)
O2	0.0990 (13)	0.1237 (15)	0.0674 (9)	0.0631 (11)	−0.0479 (9)	−0.0525 (10)
O3	0.0641 (10)	0.1039 (12)	0.0623 (9)	0.0395 (9)	−0.0222 (7)	−0.0353 (9)
C1	0.0544 (11)	0.0652 (12)	0.0471 (9)	0.0130 (9)	−0.0102 (8)	−0.0209 (8)
C2	0.0646 (13)	0.0645 (12)	0.0406 (9)	0.0096 (10)	−0.0127 (8)	−0.0191 (8)
C3	0.0590 (12)	0.0712 (13)	0.0483 (10)	0.0047 (10)	−0.0171 (8)	−0.0218 (9)
C4	0.0492 (10)	0.0690 (12)	0.0531 (10)	0.0082 (9)	−0.0114 (8)	−0.0252 (9)
C5	0.0593 (12)	0.0463 (9)	0.0488 (9)	0.0058 (8)	−0.0095 (8)	−0.0208 (7)
C6	0.0729 (13)	0.0645 (12)	0.0456 (9)	0.0192 (10)	−0.0167 (8)	−0.0267 (9)
C7	0.0737 (13)	0.0652 (12)	0.0564 (11)	0.0285 (10)	−0.0302 (9)	−0.0328 (9)
C8	0.0525 (11)	0.0648 (12)	0.0559 (10)	0.0179 (9)	−0.0197 (8)	−0.0270 (9)
C9	0.0517 (11)	0.0524 (10)	0.0455 (9)	0.0112 (8)	−0.0149 (7)	−0.0186 (8)
C10	0.0522 (11)	0.0507 (10)	0.0462 (9)	0.0083 (8)	−0.0131 (7)	−0.0197 (8)
C11	0.0675 (15)	0.131 (2)	0.0654 (13)	0.0055 (15)	−0.0253 (11)	−0.0406 (14)
C12	0.0713 (15)	0.0798 (16)	0.0950 (17)	0.0025 (12)	−0.0364 (13)	−0.0327 (13)
C13	0.0836 (17)	0.111 (2)	0.0601 (12)	0.0176 (15)	−0.0069 (11)	−0.0448 (13)
C14	0.0516 (11)	0.0654 (12)	0.0612 (11)	−0.0013 (9)	−0.0058 (8)	−0.0328 (9)
C15	0.0754 (16)	0.1040 (19)	0.0696 (14)	−0.0020 (14)	0.0076 (11)	−0.0425 (13)
C16	0.0605 (14)	0.0741 (14)	0.0950 (16)	0.0190 (11)	−0.0206 (12)	−0.0412 (13)

### Geometric parameters (Å, º)

**Table:**

O1—C2	1.369 (2)	C8—C12	1.537 (3)
O1—C13	1.421 (3)	C9—C10	1.403 (2)
O2—C7	1.224 (2)	C11—H11B	0.9600
O3—C8	1.416 (2)	C11—H11A	0.9600
O3—H3	0.87 (3)	C11—H11C	0.9600
C1—C2	1.377 (3)	C12—H12A	0.9600
C1—C9	1.389 (2)	C12—H12B	0.9600
C1—H1	0.9300	C12—H12C	0.9600
C2—C3	1.401 (3)	C13—H13B	0.9600
C3—C4	1.388 (3)	C13—H13C	0.9600
C3—C11	1.502 (3)	C13—H13A	0.9600
C4—C10	1.399 (3)	C14—C16	1.524 (3)
C4—H4	0.9300	C14—C15	1.530 (3)
C5—C6	1.343 (3)	C14—H14	0.9800
C5—C10	1.484 (2)	C15—H15A	0.9600
C5—C14	1.517 (3)	C15—H15C	0.9600
C6—C7	1.441 (3)	C15—H15B	0.9600
C6—H6	0.9300	C16—H16C	0.9600
C7—C8	1.526 (3)	C16—H16A	0.9600
C8—C9	1.510 (2)	C16—H16B	0.9600
			
C2—O1—C13	118.06 (17)	C3—C11—H11A	109.5
C8—O3—H3	109 (2)	H11B—C11—H11A	109.5
C2—C1—C9	120.19 (18)	C3—C11—H11C	109.5
C2—C1—H1	119.9	H11B—C11—H11C	109.5
C9—C1—H1	119.9	H11A—C11—H11C	109.5
O1—C2—C1	123.97 (18)	C8—C12—H12A	109.5
O1—C2—C3	114.86 (17)	C8—C12—H12B	109.5
C1—C2—C3	121.16 (17)	H12A—C12—H12B	109.5
C4—C3—C2	117.66 (18)	C8—C12—H12C	109.5
C4—C3—C11	121.55 (19)	H12A—C12—H12C	109.5
C2—C3—C11	120.78 (18)	H12B—C12—H12C	109.5
C3—C4—C10	122.77 (18)	O1—C13—H13B	109.5
C3—C4—H4	118.6	O1—C13—H13C	109.5
C10—C4—H4	118.6	H13B—C13—H13C	109.5
C6—C5—C10	119.94 (17)	O1—C13—H13A	109.5
C6—C5—C14	121.17 (16)	H13B—C13—H13A	109.5
C10—C5—C14	118.89 (16)	H13C—C13—H13A	109.5
C5—C6—C7	122.39 (17)	C5—C14—C16	109.12 (17)
C5—C6—H6	118.8	C5—C14—C15	114.28 (18)
C7—C6—H6	118.8	C16—C14—C15	109.79 (18)
O2—C7—C6	123.11 (19)	C5—C14—H14	107.8
O2—C7—C8	118.86 (19)	C16—C14—H14	107.8
C6—C7—C8	117.93 (16)	C15—C14—H14	107.8
O3—C8—C9	110.78 (15)	C14—C15—H15A	109.5
O3—C8—C7	111.14 (15)	C14—C15—H15C	109.5
C9—C8—C7	111.84 (17)	H15A—C15—H15C	109.5
O3—C8—C12	108.56 (18)	C14—C15—H15B	109.5
C9—C8—C12	107.82 (16)	H15A—C15—H15B	109.5
C7—C8—C12	106.49 (17)	H15C—C15—H15B	109.5
C1—C9—C10	120.60 (17)	C14—C16—H16C	109.5
C1—C9—C8	119.16 (17)	C14—C16—H16A	109.5
C10—C9—C8	120.17 (15)	H16C—C16—H16A	109.5
C4—C10—C9	117.56 (16)	C14—C16—H16B	109.5
C4—C10—C5	122.48 (17)	H16C—C16—H16B	109.5
C9—C10—C5	119.89 (16)	H16A—C16—H16B	109.5
C3—C11—H11B	109.5		

### Hydrogen-bond geometry (Å, º)

**Table:**

*D*—H···*A*	*D*—H	H···*A*	*D*···*A*	*D*—H···*A*
O3—H3···O2^i^	0.87 (3)	2.08 (3)	2.892 (2)	156.3
C13—H13*B*···O2^ii^	0.96	2.51	3.467 (2)	177

Symmetry codes: (i) −*x*+1, −*y*+1, −*z*+2; (ii) *x*, *y*, *z*−1.
